# Analysis of Non-Volatile Compounds in Jasmine Tea and Jasmine Based on Metabolomics and Sensory Evaluation

**DOI:** 10.3390/foods12193708

**Published:** 2023-10-09

**Authors:** Yuan Chen, Huimin An, Yiwen Huang, Jiashun Liu, Zhonghua Liu, Shi Li, Jianan Huang

**Affiliations:** 1 Key Laboratory of Tea Science of Ministry of Education, Hunan Agricultural University, Changsha 410128, China; 15773101341@163.com (Y.C.); anhuimin1995@126.com (H.A.); 19973116659@163.com (Y.H.); re13548646351@163.com (J.L.); zhonghua-liu-ms@hunan.edu.cn (Z.L.); lishidodo@163.com (S.L.); 2 National Research Center of Engineering and Technology for Utilization of Botanical Functional Ingredients, Hunan Agricultural University, Changsha 410128, China; 3 Co-Innovation Center of Education Ministry for Utilization of Botanical Functional Ingredients, Hunan Agricultural University, Changsha 410128, China; 4 Key Laboratory for Evaluation and Utilization of Gene Resources of Horticultural Crops, Ministry of Agriculture and Rural Affairs of China, Hunan Agricultural University, Changsha 410128, China

**Keywords:** Jasmine, jasmine tea, characteristic taste, non-volatile compounds

## Abstract

Scenting tea with *Jasminum sambac* is beneficial to forming a unique taste of jasmine tea, which is regulated by numerous compounds. To investigate the relationship between metabolites in jasmine and jasmine tea, as well as the impact of metabolites on the characteristic taste of jasmine tea, the liquid chromatography-mass spectrometry, sensory evaluation, and multivariate analysis were applied in this study. A total of 585 and 589 compounds were identified in jasmine tea and jasmine, respectively. After scented, jasmine tea added 70 compounds, which were believed to come from jasmine flowers. Furthermore, seventy-four compounds were identified as key characteristic compounds of jasmine tea, and twenty-two key differential metabolite compounds were believed to be used to distinguish jasmine tea scented differently and contribute to the taste of jasmine tea. Additionally, the relationship between taste compounds and aroma quality was also explored, and it was found that five compounds were positively correlated with the aroma properties of jasmine tea and seven compounds were negatively correlated with the aroma properties of jasmine tea. Overall, these findings provided insights into the future study of the mechanism of taste formation in jasmine tea and provided the theoretical basis for the production of jasmine tea.

## 1. Introduction

Jasmine tea is a type of reprocessed tea made from green tea and scented with jasmine (*J. Sambac*), which has a long history, dating back to the Song Dynasty [1]. In recent years, jasmine tea became increasingly popular due to its unique flavor and various health benefits, including effects on hypoglycemic activities, regulating immunity and antioxidant properties [2]. In accordance with the different base tea, jasmine tea can be divided into jasmine green tea, jasmine black tea, jasmine dark tea, and so on [3]. Among these scented teas, jasmine green tea, as an important branch of scented tea in China, is the most popular in both domestic and foreign markets [4,5]. This is the reason that jasmine green tea (jasmine tea in short) was selected as the main research object of this study.

Jasmine flowers belong to a temperamental type of flower, which slowly emits its fragrance as the flower buds gradually open; the scenting technology of jasmine tea is to utilize the aroma release process of jasmine and the aroma absorption process of tea, making the result of ‘tea attracts the fragrance of jasmine and enhances the flavor of tea’. As one of the raw materials for jasmine tea, the quality of jasmine flowers not only affects the aroma quality of jasmine tea, but also affects the formation and changes in taste compounds of jasmine tea during the scenting process [5,6]. According to current research, the content and composition of the aroma of jasmine tea are influenced by the origin, picking season, amount, and variety of the jasmine flower [7,8]. Nevertheless, to our knowledge, there were no studies focusing on how jasmine affects the taste quality of the jasmine tea or what compounds in jasmine contribute to it. Understanding this relationship is crucial for us to understand the mechanism of taste formation in jasmine tea and guide the improvement of the processing technology of jasmine tea products.

Taste is one of the main flavor indicators of jasmine tea, which accounts for 30% of the total score in the sensory evaluation of scented tea [9]. The coordination of taste compounds jointly forms the characteristic taste of jasmine tea. When scented tea absorbs aromas, under the action of dampness and heat, the content of bitter and astringent substances such as tea polyphenols and caffeine significantly decrease, while the content of umami-enhancing compounds such as amino acids significantly increases, and it is believed that the changes in these compounds make the taste of jasmine tea more mellow [10]. In spite of this, no study was conducted on the compounds responsible for jasmine tea’s characteristic taste systematically. Therefore, comprehensive analysis qualitatively and quantitatively of taste compounds will surely be useful to better elucidate the characteristic taste formation and retention of jasmine tea, which is helpful to guide the selection of jasmine and the production of jasmine tea products.

Metabolomics widely contributed to the identification of hundreds of different compounds in plants and other food products. In recent years, in order to understand the distinct changes in tea processing as well as their correlation with the metabolic profile and sensory quality, a wide range of key compounds were identified through metabolomics analysis [11].

Therefore, this study aimed to provide a theoretical basis for the formation of the characteristic taste of jasmine tea by identifying the key taste compounds of jasmine tea and the correlation between jasmine and jasmine tea. The taste quality of the samples was evaluated using a quantitative descriptive analysis, and ultra-high performance liquid chromatography and mass spectrometry (UPLC-Orbitrap-MS) were performed to determine the nonvolatile compounds of jasmine tea and jasmine flowers. The results will lay a foundation for the study of characteristic taste compounds of jasmine tea and provide insights and guidelines for the production of jasmine tea industry.

## 2. Materials and Methods

### 2.1. Samples Preparation

**Figure 1:**
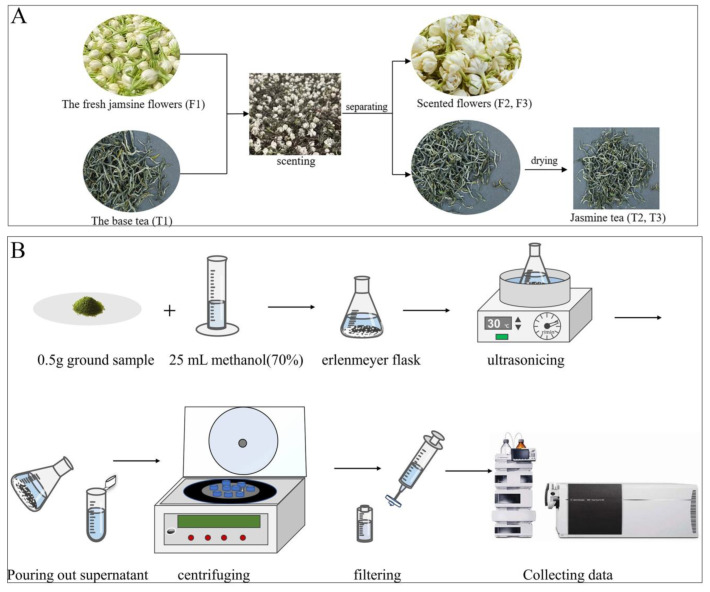
Scenting process flow plot of jasmine tea (**A**) and non-volatiles extraction of jasmine tea and jasmine infusion via LC-MS (**B**).

As a result of our collaboration with China Tea (Hunan) Co., Ltd. (Changsha, China), we were able to produce samples of jasmine tea and jasmine flowers in July 2022 and named T1, T2, T3, F1, F2, and F3. Jasmine fresh flowers (*Jasminum sambac*) were picked from Heng County, Nanning city, China. Hunan Shimen Xiefeng Famous Tea Co., Ltd. (Changde, China) provided samples of green tea (*Camellia sinensis* L.) that were used for the jasmine tea samples. T1 was the base tea that did not mix with jasmine flowers and F1 was fresh jasmine flowers that did not mix with tea. T2 and T3 are two types of jasmine tea that were scented for 12 and 18 h, respectively. F2 and F3 were jasmine flowers that were separated from T2 and T3, respectively, as soon as the scenting time reached. After the flowers were separated from the tea leaves, they were immediately stored in liquid nitrogen, and then were transferred to a refrigerator with a temperature of −80 °C for low-temperature preservation to maintain their physical and chemical properties unchanged. The scented leaves were dried at about 40 °C for 4–5 h and named jasmine tea. All the tea and flowers were divided into double; one was used for taste quality evaluation and another was used for non-volatile component detection. The samples were packaged with liquid nitrogen and dry ice, transported to the laboratory, and then stored at −80 °C until needed. The scenting processing methods were plotted in Figure 1A.

### 2.2. Sensory Evaluation Methods

To preliminarily explore the taste characteristics and potential correlation of jasmine and jasmine tea, a quantitative descriptive analysis method [12] was performed to evaluate their taste profiles, and tea infusion was prepared according to the Methodology for Sensory Evaluation of Tea [13] with slight modifications. Three grams of jasmine tea were brewed with 150 mL of boiling water for 5 min, and then the tea infusion was poured into the evaluation bowl for evaluating and scoring. Since there is no sensory evaluation standard for jasmine flowers at present, in order to correspond to the sensory evaluation of tea, the sensory evaluation method for jasmine flowers was consistency with jasmine tea. Then, the taste attributes strength of tea infusion, including floral, grassy, pungent, and astringent, were scored, respectively, from 0 to 5 (0 = none, 5 = very strong) by seven professional experts (three males and four females) that have more than 7 years of work experience on tea sensory evaluation. Finally, the average score given by these seven individuals was used to represent the intensity of each taste attribute in the samples. Each sample was evaluated three times.

### 2.3. Non-Volatile Compounds Detection Methods

#### 2.3.1. Taste Compounds Extraction

The extraction method of the jasmine tea solution was modified on the basis of GB/T8313-2008 [14]. After being ground into powder, each sample was precisely weighed at 0.5 g (to 0.0001 g). A 0.5 g sample was added to 25 mL of 70% (*v*/*v*) methanol–water extracted ultrasonically for 30 min at 30 °C and shaken every 10 min, the supernatant was poured out and centrifuged at 12,000 rcf for 10 min at 10 °C, then the extract was filtered using a 0.22 μm nylon membrane and transferred to a liquid phase vial for further detection. Quality control (QC) samples were also generated by mixing equal amounts of samples obtained throughout the analysis. Then, according to the peak area value, the Pearson correlation coefficient of the six QC samples was calculated to assess the system stability and data repeatability. Blank samples were prepared to remove the interference of background ions during detection. The methanol (MeOH) and water (LC-MS grade) were purchased from Merck (Darmstadt, Germany). Three replicates were used for each sample. The extraction process was shown in Figure 1B.

#### 2.3.2. LC-MS Condition

Orbitrap mass spectrometry technology can establish high-precision mass spectra of secondary compounds by detecting fragments without standard reagents, thus achieving preliminary screening of compounds. Compound measurements were performed using an orbital mass spectrometer and an ultra performance liquid chromatography system (UPLC Infinity 1290, Agilent Technologies, Santa Clara, CA, USA). A ZORBAX Eclipse XDB-C18 column (Agilent Technologies, 2.5 mm × 150 mm, 1.8 m) was used to separate the tea metabolome. The separation took place at 40 °C with a flow rate of 300 L/min, following a gradient elution procedure. The mobile phases consisted of 0.1% water formic acid (A) and 0.1% acetonitrile formic acid (B). The injection volume was 2 μL. The program for linear gradient elution is as follows: from 0 to 5 min, the mobile phase composition is 2% B. From 5 to 8 min, it gradually changes from 2% to 15% B. From 8 to 14 min, it further increases from 15% to 27% B. From 14 to 15 min, it continues to increase from 27% to 30% B. From 15 to 20 min, it gradually increases from 30% to 35% B. From 20 to 25 min, it increases significantly from 35% to 95% B. From 25 to 30 min, it remains at a stable 95% B. Finally, from 30 to 35 min, the mobile phase composition returns to 2% B. The linear gradient elution program adheres to the aforementioned protocol. The samples underwent separate runs in both positive and negative ionisation modes. The detailed mass parameters were as follows: sheath gas flow rate: 30 Arb, aux gas flow rate: 25 Arb, capital temperature: 350 °C, full ms resolution: 60,000, Orbitrap/MS resolution: 7500, collision energy: 10/30/60 in NCE mode, and spray voltage: 3.6 kV (positive) or—3.2 kV (negative).

### 2.4. Data Analysis Methods

The raw data files obtained by UPLC-Orbitrap/MS were first filtered and peak aligned by retention time and mass-to-charge ratio. Then, the precise molecular weight of the compound was determined by the mass-to-charge ratio in the high-resolution XIC diagram, and the formula was predicted according to the mass number deviation and adduct ion information. Finally, the compounds in the samples were identified by matching the fragment ion, collision energy, and other information of each compound in the mzCloud database. Compounds with a coefficacy of variance (CV) of <30% in the QC samples were selected as the final identification results. CD data processing software was used to integrate the chromatographic peaks detected in samples, where the peak area of each characteristic peak represented the relative quantitative value of a compound, and the total peak area was used to normalize the quantitative results.

All results recorded from three replicates are presented as mean ± SEM. In order to group samples based on the peak area value of non-volatile compounds, several analyses were conducted, including principal component analysis (PCA), hierarchical cluster analysis (HCA), partial least-squares analysis (PLS), and orthogonal partial least-squares discriminant analysis (OPLS-DA). The software used for these analyses was Simca-p (v 14.1, MKS Umetrics AB, Ume, Sweden). TB tools were used to draw a Venn plot. The additional graphs were created with Origin Pro (v 2022c, Originlab Corporation, Northampton, MA, USA) and Adobe Photoshop (v 2023, Adobe Systems Incorporated, San Jose, CA, USA).

## 3. Results and Discussion

### 3.1. Sensory Evaluation

**Figure 2:**
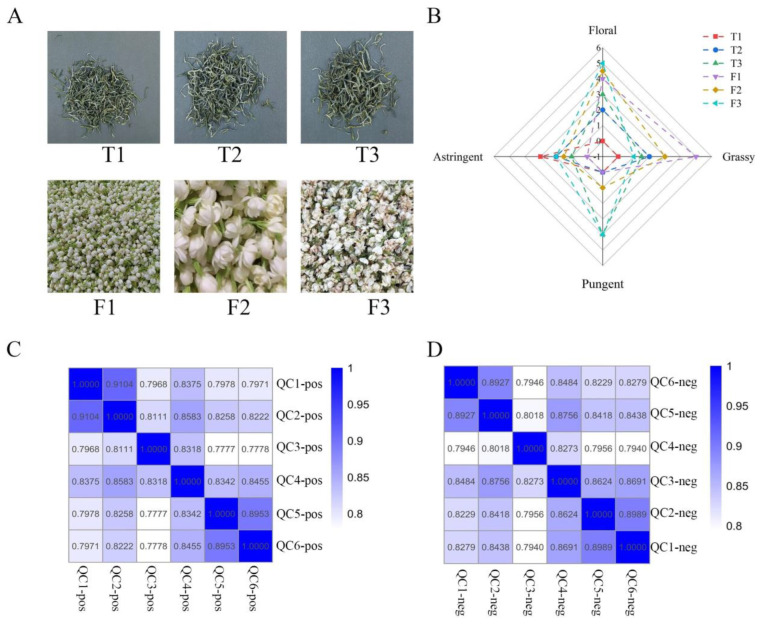
(**A**) Appearance of jasmine tea samples and jasmine samples. (**B**) Spider plot for the taste profiles of different samples. (**C**) Pearson correlation coefficient between QC samples in positive ion mode. (**D**) Pearson correlation coefficient between QC samples in negative ion mode.

The images of six samples are shown in Figure 2A. The difference in color and shape among the three types of tea are not significant because they were scented from the same base tea; this indicated that the scenting process has less impact on the color and shape of the tea, and more on the aroma and taste [15]. However, there were significant differences among the three types of jasmine flowers, F1 was in a pure white and lively state, F2 was slightly dehydrated, and F3 was in a withered and yellow state, which indicated the transition from freshness to wilting of jasmine flowers during the scenting process. Therefore, it can be inferred that the quality of jasmine is particularly important in determining the quality of jasmine tea. Four sensory attributes of samples were scored, including floral, grassy, pungent, and astringent, and their score results were plotted in Figure 2B. The sample T3 had a higher floral taste (3) than the T2 (2), but at the same time, its pungent taste is also stronger (4). The F1 with no green tea had the strongest grassy taste (5), but no pungent and astringent taste (0). The F3 had a more pronounced floral taste (5), but it was also accompanied by a stronger pungent taste (4).

The jasmine flower-splitting process was essentially an enzymatic hydrolysis process. The unopened jasmine did not have fragrance, and they could only be scented when the jasmine flower buds bloomed to about 90% [16,17]. At this time, the fragrance concentration of jasmine flowers just began to increase. With the increase in scenting time and temperature, the speed of enzyme catalysis accelerated, which accelerated the release of aroma substances, thus increasing the fragrance concentration [18]. However, when the scenting reached a certain time, jasmine flowers transitioned from fresh to withered, and the freshness and concentration of the aroma decreased, while the pungent aroma increased. The sensory evaluation results of jasmine are consistent with the changes in jasmine aroma during the scenting process, which indicated that these volatile compounds might dissolve in the jasmine infusion when jasmine flowers were brewed, thereby affecting the taste of jasmine. It was worth noting that the taste intensities of jasmine flowers were generally consistent with the taste intensities of their scented tea; this result indicates that there was a connection between the taste of jasmine tea and jasmine flowers. Furthermore, different scenting technologies resulted in jasmine tea and jasmine flowers with different intensities of four taste characteristics, and tea and jasmine with longer scenting time (T3 and F3) tended to have a stronger unpleasant taste. Therefore, it is worth further exploring the optimal scenting time for jasmine tea with the best taste quality.

### 3.2. Identification and Quantification of the Non-Volatile Compounds by UPLC-Orbitrap-MS

**Figure 3:**
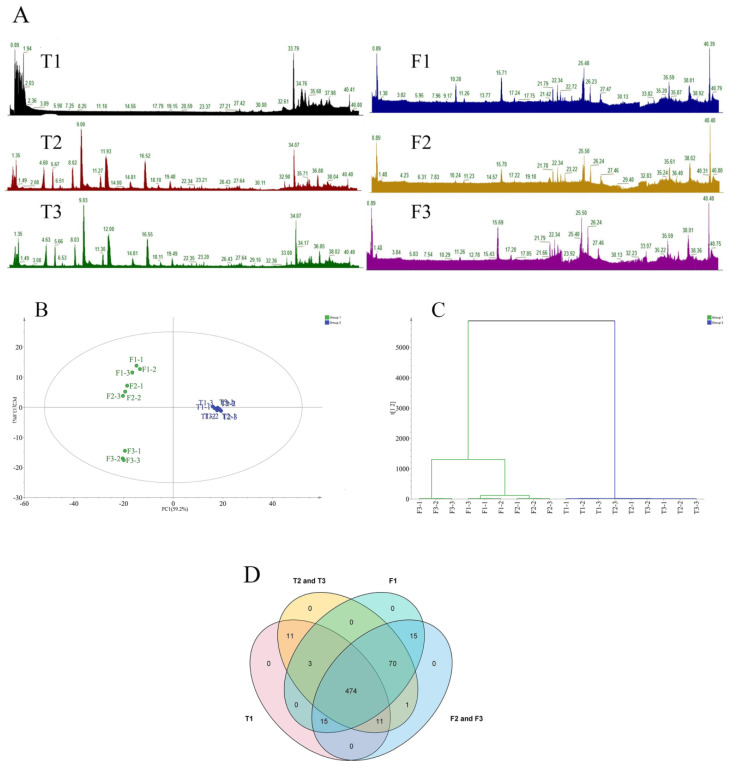
(**A**) The total ion chromatography (TIC) plots of six samples. (**B**) PCA analysis of the nonvolatile compounds in jasmine samples and tea samples. (**C**) HCA analysis of the nonvolatile compounds in jasmine samples and tea samples. (**D**) The Venn plot of the quantity of compounds contained in each group.

The quality control results show that the instrument analysis system of this experiment was stable and the experimental data were reliable (Figure 2C,D), which provides assurance for the subsequent data analysis. The nonvolatile compounds of samples were subjected to UPLC-Orbitrap-MS analysis (Figure 3A). A total of 2682 ion features (negative model) and 1944 ion features (positive model) were observed in jasmine and jasmine tea. Finally, 585 compounds and 589 compounds were identified in the jasmine tea and jasmine, respectively, according to authentic standards. The figure shows that the total content of nonvolatile compounds in jasmine flowers were much higher than those in tea, and the content and types of nonvolatile compounds in base tea (T1) were significantly different from jasmine tea (T2 and T3).

### 3.3. The Taste of Jasmine and Jasmine Tea Was Obviously Different

PCA and HCA were employed to compare and contrast the six samples by using the peak areas of 600 non-volatile compounds obtained through UPLC-Orbitrap/MS. The results of the PCA analysis show that the first two principal components explained 59.2% and 13.8% of the total variance, respectively (R2X = 0.73) (Figure 3B). The results of the HCA tree structure (Figure 3C) are in agreement with those of the PCA: The six samples fell clearly into two major categories: “jasmines” and “teas”. These results suggest that the taste difference between jasmine and tea was obvious. Overall, the subsequent analysis was based directly on these results.

### 3.4. Comparative Analysis of the Nonvolatile Compounds of Jasmine and Jasmine Tea Samples

The Venn plots depicted the number of nonvolatile compounds that samples shared or were unique. By comparing the nonvolatile compounds of non-scented jasmine (F1) and tea (T1), we found that they shared 492 nonvolatile compounds and had 85 and 22 unique nonvolatile compounds, respectively (Figure 3D). Among these 85 compounds, 70 of them were present in scented tea (T2 and T3), but not in base tea (T1), which indicated that these compounds may be brought into tea by jasmine flowers during the scenting process. More interestingly, 11 of these 22 compounds unique to T1 were also detected in the scented jasmine flowers (F2 and F3), which indicated that some non-volatile compounds in jasmine and jasmine tea were transferring to each other during the scenting process. This indicates that the quality of jasmine is also very important for the formation of the taste of jasmine tea. In future study, it is worth further exploring how these nonvolatile compounds transfer and what role these compounds play in taste, which will provide a theoretical basis for the selection of jasmine flowers when processing jasmine tea.

By comparing the base tea (T1) and scented teas (T2 and T3), we found that they shared 499 compounds, and had 15 and 71 unique compounds, respectively. By comparing the F1 and scented jasmine flowers (F2 and F3), we found that they shared 574 compounds, and had 3 and 12 unique compounds, respectively. The 15 and 3 compounds, which only existed in non-scented samples (T1 and F1) and were not present in scented samples (T2, T3, F2, and F3), may be transformed into other compounds or degraded during the scenting process, or may be masked by other compounds with high concentrations. This result indicates that the scenting process was not only a simple physical adsorption, but also accompanied by chemical reactions. This result was the same as the generally recognized aroma absorption mechanism of jasmine tea [19], which may promote further development of a formation mechanism of the characteristic taste of jasmine tea products.

### 3.5. Detection of the Transformation of Key Non-Volatile Compounds in Jasmine Tea

**Figure 4:**
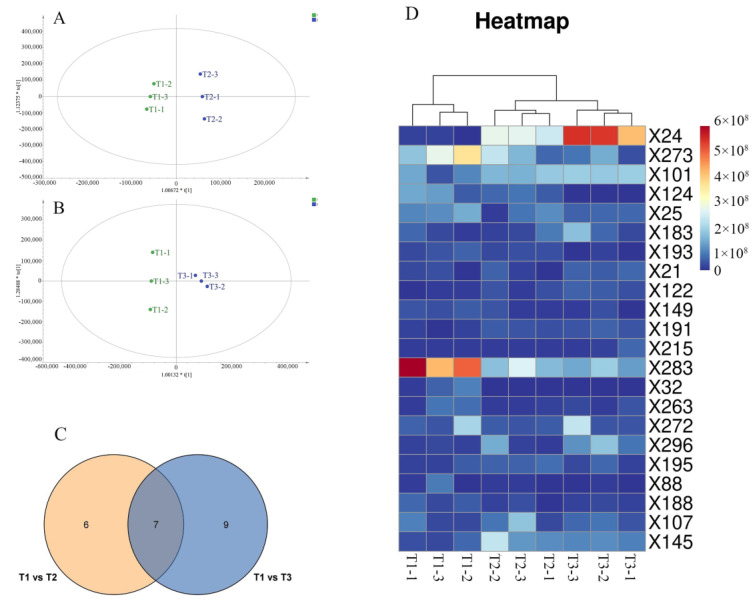
(**A**) OPLS-DA of tea (*Camellia sinensis*) samples treated with no scenting (T1) and T2; (**B**) OPLS-DA of tea (*Camellia sinensis*) samples treated with no scenting (T1) and T3; (**C**) Venn plot of differential compounds in three tea samples; and (**D**) a heatmap of 22 identified differential compounds in the T1, T2, and T3.

In order to fully study the reasons for the taste changes after scenting, PCA and HCA were applied to the metabolite data obtained after purification and UV scaling. The (PCA) plots (Figure S1A) showed significant differences between base tea (T1) and scented tea (T2, T3), which indicated that the scenting changed the non-volatile compounds of the base tea a lot. At the same time, the samples T2 and T3 were also clearly distinguished, indicating that the metabolites are affected differently by the duration of scenting. HCA indicated a comparable clustering effect to PCA (Figure S1B). To identify differences in metabolites caused by scenting, a supervised OPLS-DA analysis of metabolite data for the T1 versus T2 and the T1 versus T3 was performed (Figure 4A,B). The cross-validation analysis including 200 permutation assessments showed that the OPLS-DA models were reliable, with R2 intercepts of 0.432 and 0.335, respectively, and Q2 values of −1.08 and −1.07 (refer to Figure S1C,D). Using a variable importance in projection value >1 in the T1 versus T2 and the T1 versus T3 comparisons, 64 and 44 metabolites were considered key characteristic compounds of jasmine tea, respectively. The information of the key characteristic compounds was shown in Supplementary Table S1. The multiple of difference (FoldChange, FC) was the ratio of the mean of all biological repeated quantitative values of each metabolite in the comparison group. A total of 13 and 16 compounds were identified as potential differential metabolites on the basis of a variable importance in projection value > 1 and FC > 2 or FC < 0.5 in the T1 versus T2 and the T1 versus T3 comparisons. Using these data, we constructed a Venn diagram (Figure 4C) and identified a combined total of 22 potentially differential metabolites: eight organic acids (X25, X21, X193, X101, X183, X107, X88, and X296), one flavonoid and their glycosides (X272), three lipids (X215, X124, and X188), three terpenoids (X24, X191, and X32), three heterocyclic compounds (X145, X273, and X263), and four others (X149, X283, X195, and X122). Of these 22 metabolites, 7 were common and 6 and 9 were specific for T2 and T3, respectively. This finding indicated that T3 underwent a greater number of chemical alterations compared to T2, potentially attributed to the extended duration of scenting.

Flavonoids and their glycosides, which possess strong antioxidative bioactivity and potential benefits for the cardiovascular system, play an important role in forming the astringency of tea leaves [20]. In this study, the levels of isoscutellarein (X272) decreased significantly during the scenting process by 57.65% and 60.67% in T2 and T3, respectively, compared with this in the initial samples (T1). The decrease in flavonoids in T3 was greater than that in T2, which may be due to the prolongation of the scenting time, thereby prolonging the degradation time of flavonoids. Flavonols and flavones contribute significantly to the astringency and bitterness of tea soup and can also increase the bitterness of caffeine [21,22]. Thus, the degradation of flavonols and flavones during the scenting process may be the reason why the astringency of tea leaves decreased after being scented, which may play a key role in the formation of the characteristic taste of jasmine tea. In addition, among these differential compounds, o-methyl anthranilate (X122) is considered a compound with a pungent taste [23], and the content of o-methyl anthranilate in these three types of tea is: T3 > T2 > T1, which may explain the reason for the strong pungent taste of T3. It is worth noting that the content of o-methyl anthranilate is significantly higher in F3 than in F2. These results are consistent with the sensory evaluation results. Figure 4D depicts the heatmap created to analyze the disparities in these crucial metabolites among T1, T2, and T3, which shows that the contents of these metabolites in the T1 significantly differed from those in T2 or T3. These differential metabolites potentially contributed to the characteristic taste of jasmine tea.

**Figure 5:**
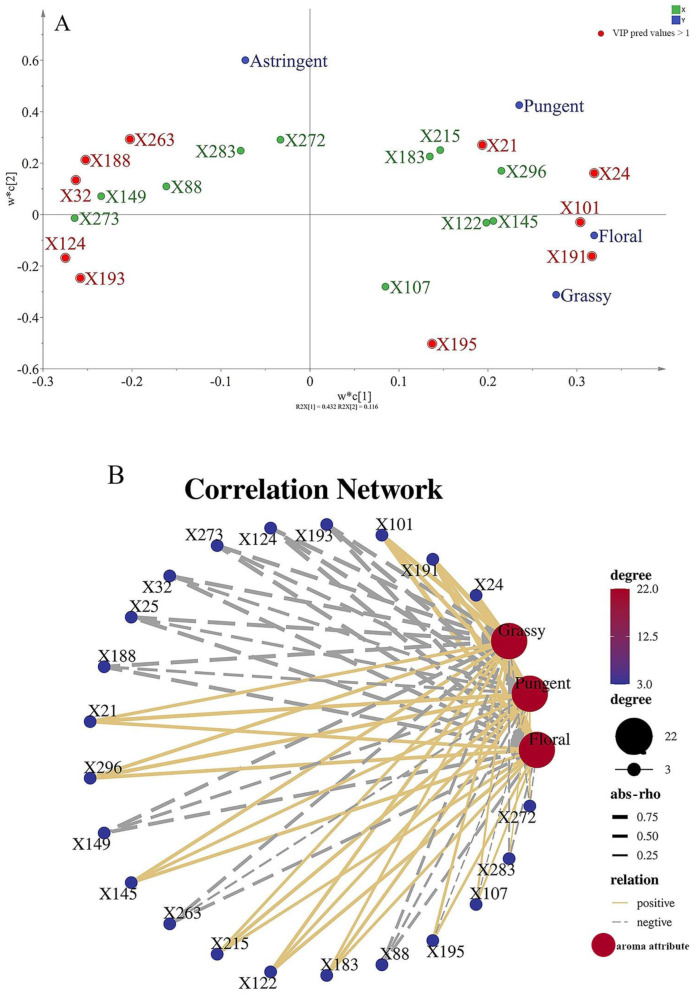
(**A**) PLS analysis of jasmine tea quality and chemical compounds. (**B**) The correlation network between aroma attributes and differential compounds of jasmine tea.

To investigate the relationship between the taste attributes and these differential metabolites, four typical taste attributes in jasmine tea, astringent, pungent, floral, and grassy, were used to establish a PLS model. As presented in Figure 5A, based on the VIP pred values >1, the intensities of these characteristics showed a positive correlation with the levels of jasmonic acid (X21), guanidinoacetic acid (X193), 3-(3,4-Dihydroxy-5-methoxy)-2-propenoic acid (X101), acetoacetic acid (X124), narirutin (X195), galactosylsphingosine (X188), gliquidone (X263), betulin (X32), gamma-terpinene (X24), 1,4-Dimethyl-7-ethylazulene (X191).

### 3.6. Correlation Analysis of Aroma and Taste Compounds

From the aroma sensory evaluation results of jasmine tea, including floral, grassy, and pungent odor, we found that it was consistent with the taste results. The average score for each attribute are shown in Supplementary Table S2. As shown in Figure 5B, five differential compounds were positively correlated with the aroma properties of jasmine tea and seven differential compounds were negatively correlated with the aroma properties of jasmine tea, which may be caused to the following reasons. On the one hand, the solubility of aroma compounds in tea soup affects the taste. It is worth noting that some metabolites detected in jasmine tea are volatile compounds, for example, gamma-terpinene (X24) was considered as a special aroma of shuixian fresh leaves [24], O-methyl anthranilate (122) was considered as the main volatile component of jasmine flowers [25], and jasmone (X26) was considered to have jasmine fragrance [26]. Therefore, it can be speculated that some volatile components were indeed dissolved in the tea infusion and the content and proportion of these volatile compounds in turn had a key impact on the taste of the jasmine tea. On the other hand, some taste compounds may be odor precursor components; this result can provide reference for further research on the aroma quality chemistry of jasmine tea in the future. However, in this study, not all the volatile compounds dissolved in the tea soup were detected. One reason is that the volatile compounds will evaporate during the process of extraction and detection, resulting in a decrease in the content of compounds dissolved in the tea soup and then cannot be detected. Another reason may be related to the limited detection method. Liquid chromatography-mass spectrometry technology is mainly used to detect non-volatile compounds, and relying solely on this technology, it is difficult to achieve comprehensive detection.

In recent years, many studies explored the impact of taste compounds on aroma. Green et al. found that NaCl and citric acid can significantly enhance the vanilla sweetness, lemon fruit aroma, and furan burnt aroma properties of the mixed solution system of vanillin, citral, and furanone [27]. Furthermore, the addition of sucrose can significantly enhance the post-nasal aroma perception of beverages and cream desserts, and enhance the intensity of the “cherry” and “vanilla” flavor properties [27]. This type of phenomenon is called cross modal perceptual interaction, which refers to a sensory form (such as olfactory) stimulus that can compensate for or satisfy desires related to another sensory form (such as taste) [28]. This may also be the reason for the correlation between taste and aroma. However, the aroma and taste components of tea and jasmine are complex, and the interaction between odor and taste is needed to be further researched. In summary, these results indicate that the taste and quality of jasmine tea are also influenced by volatile compounds.

## 4. Conclusions

In this study, 600 compounds in jasmine flowers and jasmine teas were identified using UPLC-Orbitrap-MS analysis. Jasmine tea added 70 compounds during the scenting process that were common to jasmine flowers and were believed to come from jasmine flowers. This result indicates that the metabolites in jasmine flowers affect the formation of taste quality of tea and provides a theoretical basis for the selection of jasmine flowers in the future jasmine tea processing process. The results of metabolomics combined with sensory evaluation show that the taste quality of jasmine tea scented for 12 h was better than that of jasmine tea scented for 18 h. Of the 600 compounds, a total of 74 compounds were considered to be the key characteristic compounds of jasmine tea and the contents of 22 exhibited sharp fluctuations during the scenting process. Furthermore, some key differential characteristic taste compounds were significantly correlated with the aroma attributes. In conclusion, the obtained results will provide new insights for the study of characteristic taste compounds in jasmine tea, and provide potential biomarkers for production and quality control of the jasmine tea industry.

## Data Availability

Data will be made available on request.

## Supplementary Materials

The following supporting information can be downloaded at: https://www.mdpi.com/article/10.3390/foods12193708/s1, Figure S1: Multivariate analysis of tea (*Camellia sinensis*) products processed with no scenting (T1) and scenting (T2 and T3), Table S1: Information of the key characteristic compounds in jasmmine tea, Table S2: Aroma scores of samples.
