# Supplementary material for: Analysis of Non-Volatile Compounds in Jasmine Tea and Jasmine Based on Metabolomics and Sensory Evaluation
Source: Foods. 2023 Oct 9;12(19):3708. doi: 10.3390/foods12193708 (PMC10572636; doi:10.3390/foods12193708)

Table S1:

Information of the key characteristic compounds in jasmmine tea

Tentative Identification	No.	m/z	RT(min)	Formula	CAS	VIP	Flavor descriptor
Heterocyclic Compounds							
Catechin	X159	289.072	7.79	C15H14O6	154-23-4	6.10885	
Cyanuric acid	X273	128.01	11.45	C3H3N3O3	108-80-5	3.84875	Slightly bitter
indoline	X282	120.081	6.07	C8H9N	496-15-1	2.5517	
Gliquidone	X263	526.203	9.02	C27H33N3O6S	33342-05-1	2.18812	
(1H-Indol-3-yl)(2,2,3,3-tetramethylcyclopropyl)methanone	X292	242.158	2.58	C16H19NO	895152-66-6	1.42313	
Amino Acidsand their derivatives							
L-Isoleucine	X271	132.066	2.98	C6H13NO2	443-79-8	4.20053	Bitter
L-Valine	X45	118.086	2.30	C5H11NO2	72-18-4	7.06329	
L-Lysine	X47	129.102	1.77	C6H14N2O2	56-87-1	3.9618	Bitter
Dimethylglycine	X125	104.071	2.24	C4H9NO2	1118-68-9	2.24031	
L-Arginine	X213	175.119	1.44	C6H14N4O2	74-79-3	1.99798	
2-Aminoisobutyric acid	X44	104.071	5.39	C4H9NO2	62-57-7	1.91651	
2-(Methylamino)benzoic acid	X296	152.07	11.95	C8H9NO2	119-68-6	1.75881	
N-Alpha-acetyllysine	X224	188.07	6.72	C8H16N2O3	1946-82-3	1.67215	
L-Glutamine	X115	146.081	1.60	C5H10N2O3	56-85-9	1.64328	
Guanidoacetic acid	X193	117.055	2.26	C3H7N3O2	352-97-6	1.27986	
Organic acid							
Jasmonic Acid	X21	211.132	0.54	C12H18O3	6894-38-8	1.47566	
Pipecolic acid	X46	130.05	2.43	C6H11NO2	535-75-1	5.14305	
Isochlorogenic acid b	X57	515.119	9.22	C25H24O12	14534-61-3	3.67118	
3-(3,4-Dihydroxy-5-methoxy)-2-propenoic acid	X101	211.06	5.17	C10H10O5	110642-42-7	2.98238	
3,4-Dihydroxybenzeneacetic acid	X206	168.042	1.39	C8H8O4	102-32-9	2.38647	
O-Methyl anthranilate	X122	152.07	13.59	C8H9NO2	134-20-3	1.57832	Pungent
Succinic acid semialdehyde	X107	101.023	1.87	C4H6O3	692-29-5	1.13965	
trans-Cinnamate	X167	131.049	8.82	C9H8O2	140-10-3	1.07538	
Terpenoid							
Gamma-terpinene	X24	137.132	13.60	C10H16	99-85-4	4.77532	
Pulegone	X23	153.127	7.78	C10H16O	89-82-7	2.62635	
(2E)-2-(hydroxymethyl)-3-[3-oxo-5-(propan-2-yl)-1,3,4,5,6,7-hexahydro-2-benzofuran-4-yl]prop-2-enoic acid	X312	279.124	9.46	C15H20O5		2.36761	
Betulin	X32	443.387	13.38	C30H50O2	473-98-3	2.32406	
1,4-Dimethyl-7-ethylazulene	X191	167.118	0.57	C14H16	529-05-5	1.58385	
Alkaloids							
7-Methylxanthine	X235	147.03	5.60	C6H6N4O2	552-62-5	1.41064	
Theobromine	X200	181.071	13.34	C7H8N4O2	83-67-0	8.08453	Bitter
1-Methylxanthine	X220	167.056	5.20	C6H6N4O2	6136-37-4	1.31789	
Sanguinarine	X54	333.095	6.75	C20H14NO4	2447-54-3	1.14211	

Lipids							
Suberic acid	X215	173.081	6.73	C8H14O4	505-48-6	1.09389	
Jasmone	X29	165.127	13.44	C11H16O	488-10-8	1.02885	Woody, bitter
(Z)-4-Hydroxy-6-dodecenoic acid lactone	X60	177.038	12.71	C6H10O6	18679-18-0	1.33117	
Galactosylsphingosine	X188	461.341	12.98	C24H47NO7	2238-90-6	1.31691	
Stearidonic acid	X91	259.206	13.81	C18H28O2	20290-75-9	1.36217	
Acetoacetic acid	X124	103.039	0.54	C4H6O3	541-50-4	1.33988	
Flavonoids and their glycosides							
Kaempferol 3-sophorotrioside	X227	773.222	8.15	C33H40O21	80714-53-0	4.865	
Afzelechin	X214	275.091	8.49	C15H14O5	2545-00-8	3.51076	
Isoscutellarein	X272	287.054	9.94	C15H10O6	41440-05-5	2.08123	
Kaempferitrin	X209	577.157	7.08	C27H30O14	482-38-2	1.23048	
Luteolin	X162	286.043	11.84	C15H10O6	491-70-3	1.12786	
Aromadendrin	X133	288.058	8.51	C15H12O6	480-20-6	1.10258	
Eriodictyol	X123	287.056	10.81	C15H12O6	552-58-9	1.03301	
Carbohydrates and carbohydrate derivatives							
Thermopsoside, crotonoyl	X2	530.141	11.93	C26H26O12		1.23049	
L-Arabinose	X19	131.035	13.85	C5H10O5	87-72-9	1.14298	
Mannitol	X205	182.081	5.76	C6H14O6	69-65-8	1.55395	Sweet
Galactose 1-phosphate	X291	241.011	1.55	C6H13O9P	2255-14-3	1.33497	
2',3,4,4',6'-Peptahydroxychalcone 4'-O-glucoside	X42	451.137	12.17	C21H22O11		1.1591	
Phenols							
Gentisate aldehyde	X148	137.024	13.87	C7H6O3	1194-98-5	1.37906	
Schizandrin	X183	431.215	13.85	C24H32O7	7432-28-2	1.32567	
Rosmarinic acid	X180	359.134	8.58	C18H16O8	20283-92-5	1.20762	
Piceatannol	X77	225.058	11.90	C14H12O4	10083-24-6	2.9171	
Others							
4-Hydroxybenzoate	X143	137.022	8.71	C7H6O3	456-23-5	1.22008	
trans-3,4-Difluorocinnamic acid	X99	184.033	7.49	C9H6F2O2	112897-97-9	3.18789	
4-Hydroxy-3-(3-methyl-2-butenyl)acetophenone	X149	187.108	1.69	C13H16O2	26932-05-8	1.33391	
Hispidulin acetate	X286	425.088	9.48	C22H18O9	1178-23-0	6.2161	
l-(+)-Rhamnose monohydrate	X283	181.068	11.83	C6H14O6	10030-85-0	5.55958	
Neoglucobrassicin	X145	478.072	8.82	C17H22N2O10S2	5187-84-8	3.06432	
Kojic acid	X25	143.034	2.23	C6H6O4	501-30-4	2.1191	
3-Methyl-1-(2,4,6-trihydroxyphenyl)-1-butanone	X222	211.097	8.23	C11H14O4	26103-97-9	2.01478	
N-Carbamoylputrescine	X105	132.102	9.01	C5H13N3O	6851-51-0	1.924	
Violanthin	X208	578.164	7.44	C27H30O14	40581-17-7	1.84346	
4-Hydroxy-3-(3-methyl-2-buten-1-yl)phenyl 6-O-[(2R,3R,4R)-3,4-dihydroxy-4-(hydroxymethyl)tetrahydro-2-furanyl]-beta-D-glucopyranoside	X302	471.187	8.96	C22H32O11		1.64882	

Narirutin	X195	579.175	1.63	C27H32O14	14259-46-2	1.52063	
Choline	X64	104.107	7.31	C5H14NO	62-49-7	1.40537	
Citric acid	X68	190.927	3.10	C6H8O7	77-92-9	1.31941	Sweet
Cyanidin 3-(2G-glucosylrutinoside)	X192	757.222	8.38	C33H41O20	55028-57-4	1.28468	
Hydroxyphenyllactic acid	X88	183.077	0.54	C9H10O4	306-23-0	1.31752	
Epoxy Fluor 7	X1	390.138	5.01	C23H19NO5	863223-43-2	1.1538	
Uridine 5'-triphosphate tris salt	X4	467.134	11.17	C9H15N2O15P3	108321-53-5	1.04578	
Mudanpioside A	X76	613.192	7.19	C31H34O13		1.03987	
Tyrosol	X196	138.066	6.03	C8H10O2	501-94-0	1.08951	

Table S2:

Aroma scores of samples.

Samples	Floral	Grassy	Pungent
T1	0	0	0
T2	2	1	1
T3	3	3	4.5
F1	4	5	1
F2	4.5	4	3.5
F3	5	2	5

Figure S1. Multivariate analysis of tea (*Camellia sinensis*) products processed with no scenting (T1) and scenting (T2 and T3). (A) Principal component analysis (PCA) score plot; (B) Dendrogram plot obtained through PCA; (C) Permutation plot of orthogonal partial least-squares discriminant analysis (OPLS-DA) with T2 versus T1 (intercepts of R^2 and Q^2 being 0.432 and −1.08, respectively); and (D) permutation plot of OPLS-DA with T3 versus T1 (intercepts of R^2 and Q^2 being 0.335 and −1.07, respectively).

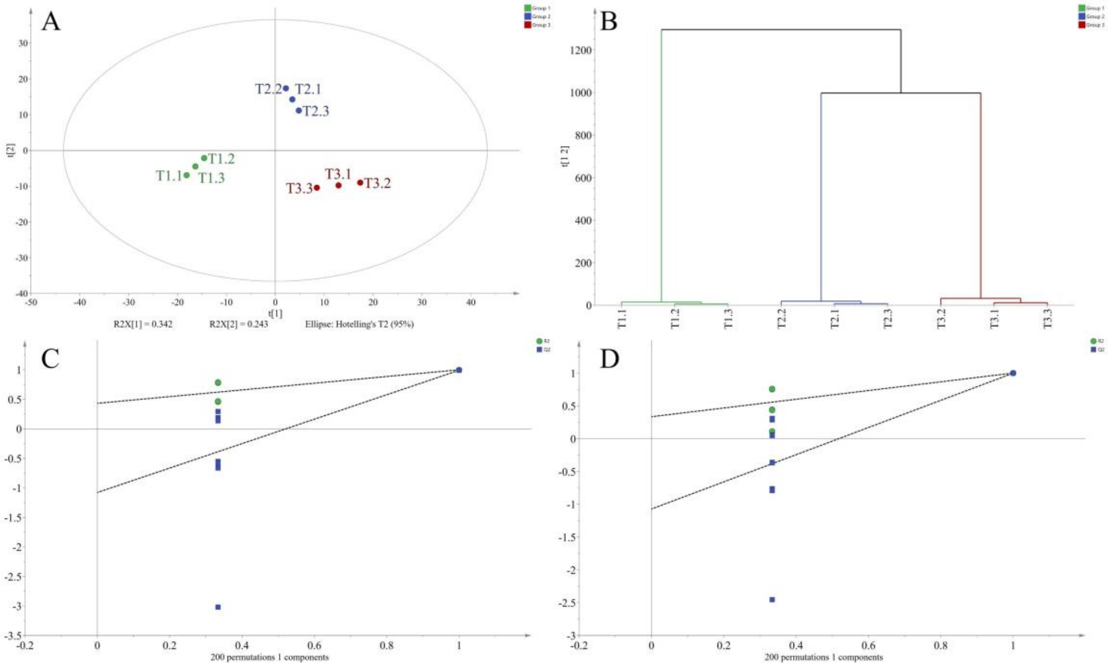

Supplement: Supplementary file 1 [file foods-12-03708-s001.zip › foods-2620484-supplementary.pdf]
